# Supplementary material for: Complex spike synchrony dependent modulation of rat deep cerebellar nuclear activity
Source: eLife. 2019 Jan 9;8:e40101. doi: 10.7554/eLife.40101 (PMC6326725; doi:10.7554/eLife.40101)
Supplement: Transparent reporting form [file elife-40101-transrepform.pdf]

eLIFE
1st Floor
24 Hills Road
Cambridge CB2 1JP, UK
P 01223 855340
W elifesciences.org
T @elife

eLife's transparent reporting form

We encourage authors to provide detailed information *within their submission* to facilitate the interpretation and replication of experiments. Authors can upload supporting documentation to indicate the use of appropriate reporting guidelines for health-related research (see [EQUATOR Network](#)), life science research (see the [BioSharing Information Resource](#)), or the [ARRIVE guidelines](#) for reporting work involving animal research. Where applicable, authors should refer to any relevant reporting standards documents in this form.

If you have any questions, please consult our Journal Policies and/or contact us: editorial@elifesciences.org.

Sample-size estimation

- You should state whether an appropriate sample size was computed when the study was being designed
- You should state the statistical method of sample size computation and any required assumptions
- If no explicit power analysis was used, you should describe how you decided what sample (replicate) size (number) to use

Please outline where this information can be found within the submission (e.g., sections or figure legends), or explain why this information doesn't apply to your submission:

The cells used for this study are a subset of a larger population that had been obtained for a previous study. Given the difficulty in meeting the inclusion criteria for the present work (e.g., at least four Purkinje cells in crus 2a showing evidence of a monosynaptic connection to the simultaneously recorded cerebellar nuclear cell), all experiments in which the criteria were clearly met were included. The overall population, N's for the subset of recordings for this study, and the inclusion criteria are described in the first paragraph of the results section.

Replicates

- You should report how often each experiment was performed
- You should include a definition of biological versus technical replication
- The data obtained should be provided and sufficient information should be provided to indicate the number of independent biological and/or technical replicates
- If you encountered any outliers, you should describe how these were handled
- Criteria for exclusion/inclusion of data should be clearly stated
- High-throughput sequence data should be uploaded before submission, with a private link for reviewers provided (these are available from both GEO and ArrayExpress)

Please outline where this information can be found within the submission (e.g., sections or figure legends), or explain why this information doesn't apply to your submission:

eLife Sciences Publications, Ltd is a limited liability non-profit non-stock corporation incorporated in the State of Delaware, USA, with company number 5030732, and is registered in the UK with company number FC030576 and branch number BR015634 at the address 1st Floor, 24 Hills Road, Cambridge CB2 1JP | August 2014

-How often each experiment was performed is included in the results subsections for each set of results.
-There is only biological replication (of cell groups across animals); technical replication is not relevant.
-N values are given for every experiment and analysis in the text.
-No outliers were excluded; however, not every experiment could be used for all analyses. Which experiments were used for each analysis is stated in all cases, and when an experiment was excluded from a particular analysis the reason for doing so is given (e.g., the phenomenon being analyzed was not present in that experiment).
-Criteria for exclusion/inclusion are stated in the text.
High-throughput sequence data: NA

Statistical reporting

- Statistical analysis methods should be described and justified
- Raw data should be presented in figures whenever informative to do so (typically when N per group is less than 10)
- For each experiment, you should identify the statistical tests used, exact values of N, definitions of center, methods of multiple test correction, and dispersion and precision measures (e.g., mean, median, SD, SEM, confidence intervals; and, for the major substantive results, a measure of effect size (e.g., Pearson's r, Cohen's d)
- Report exact p-values wherever possible alongside the summary statistics and 95% confidence intervals. These should be reported for all key questions and not only when the p-value is less than 0.05.

Please outline where this information can be found within the submission (e.g., sections or figure legends), or explain why this information doesn't apply to your submission:

-Standard statistical tests for assessing the significance of means and correlation coefficients were used, and are taken from Zar (1999, Biostatistical Analyses). This is stated in the Data Analysis subsection of the Materials and Methods section.
-The statistical analysis of correlograms for identifying synaptic connectivity is described in the Materials and Methods subsection entitled 'Identification of monosynaptically-connected PC-DCN cell pairs.'.
-Raw data is given to show the quality of the cerebellar nuclear cell recordings.
-The exact p-values are given for all statistical tests, except in cases where a set of experiments were tested and all failed to show a significant result. In these cases, the lack of significance is summarized as, p < .05.

(For large datasets, or papers with a very large number of statistical tests, you may upload a single table file with tests, Ns, etc., with reference to sections in the manuscript.)

Group allocation

- Indicate how samples were allocated into experimental groups (in the case of clinical studies, please specify allocation to treatment method); if randomization was used, please also state if restricted randomization was applied
- Indicate if masking was used during group allocation, data collection and/or data analysis

Please outline where this information can be found within the submission (e.g., sections or figure legends), or explain why this information doesn't apply to your submission:

NA

Additional data files ("source data")

- We encourage you to upload relevant additional data files, such as numerical data that are represented as a graph in a figure, or as a summary table
- Where provided, these should be in the most useful format, and they can be uploaded as "Source data" files linked to a main figure or table
- Include model definition files including the full list of parameters used
- Include code used for data analysis (e.g., R, MatLab)

- Avoid stating that data files are "available upon request"

Please indicate the figures or tables for which source data files have been provided:

None
